# Job vacancies at the European Centre for Disease Prevention and Control

**DOI:** 10.2807/1560-7917.ES.2017.22.32.30590

**Published:** 2017-08-10

**Authors:** Eurosurveillance Editorial team

**Affiliations:** 1 European Centre for Disease Prevention and Control (ECDC), Stockholm, Sweden

The European Centre for Disease Prevention and Control (ECDC) invites applications for the following positions:

Head of Disease Programme Emerging and Vector-borne Diseases
Expert Medical Entomology
Expert Infectious Diseases Epidemiology
Scientific Officer Surveillance

The deadlines for the applications vary. For more information, please visit the ECDC website: http://ecdc.europa.eu/en/aboutus/jobs/Pages/JobOpportunities.aspx

